# The Prevalence of Depression and Anxiety and Its Association with Sleep Quality in the First-Year Medical Science Students

**DOI:** 10.1155/2024/7102081

**Published:** 2024-04-15

**Authors:** Maryam Garmabi, Zahra Andishmand, Fatemeh Naderi, Ahmad Sharifnezhad, Fatemeh Darrudi, Roghayeh Malekzadeh, Asieh Amini, Ali Gholami

**Affiliations:** ^1^Student Counseling Center, Neyshabur University of Medical Sciences, Neyshabur, Iran; ^2^Department of Basic Sciences, School of Medicine, Neyshabur University of Medical Sciences, Neyshabur, Iran; ^3^Noncommunicable Diseases Research Center, Neyshabur University of Medical Sciences, Neyshabur, Iran; ^4^Ph.D. Candidate in English Language Teaching, Razi University, Kermanshah, Iran; ^5^Department of Epidemiology and Biostatistics, School of Public Health, Neyshabur University of Medical Sciences, Neyshabur, Iran

## Abstract

**Methods:**

A total number of 471 freshmen students (NUMS) participated in the study, pinpointing that the data are collected in 2019 and 2020. In line with measuring depression, anxiety, and sleep quality, the Persian Beck Depression Inventory-II, Beck Anxiety Inventory, and Pittsburgh Sleep Quality Index were employed. The associations between depression and anxiety with sleep quality were assessed by using the multiple logistic regression model. All statistical analyses were conducted in STATA^14^, and the significant level was set at *P* < 0.05.

**Results:**

The prevalence of depression, anxiety, and poor sleep quality in the study population was 21.4%, 31.9%, and 28%, respectively. Analytical analyses indicated that after adjusting for studied covariates, the odds of poor sleep quality in individuals with depression were 3.5 times higher compared to the counter group (*P* < 0.001). Moreover, the odds of poor sleep quality in individuals with anxiety were 2.1 times higher compared to the counter group (*P* < 0.001).

**Conclusion:**

Noticeable proportion of freshmen students suffer from depression and anxiety; in line with such a critical issue, our study found that depression and anxiety had a statistical association with sleep quality in study population. From this respect, it seems that providing essential interventions and psychological counseling services could be constructive for the freshmen medical students.

## 1. Introduction

Currently, up surging concerns have been garnered with regard to mental disorders such as depression, anxiety, and stress at the global level [1, 2]. Young individuals, particularly freshmen students, are more likely prone to various types of mental disorders [3]. Entering to new locations and experiencing new residency (dormitory life), having new food patterns, meeting new friends, and a wide range of uncertainties lead them to experience conditions of depression and anxiety, besides posing them to risk of emotional and mental vulnerabilities [4–6].

University students are one of the most dynamic pillars of society's sociocultural structure. Based on the previous epidemiological literature, a remarkable proportion of university students are suffering from depression and anxiety [7–9]. Concerning depression in students, its prevalence has changed in different contexts such as 68.5% in Hong Kong [10], 43.7% in India [11], 37% in Malaysia [12], 43% in Saudi Arabia [13], 63.3% in Egypt [6], and 27.1% in Turkey [14]. Similarly, the prevalence of anxiety among students in Hong Kong [10], India [11], Malaysia [12], Saudi Arabia [13], Egypt [6], and Turkey [14] were 54.4%, 68.6%, 63%, 63%, 78.4%, and 47.1%, respectively. More to say, research has revealed that sleep is inexorably associated with both physiological and psychological well-being [15–17]. Sleep disruption hurts psychosocial health, performance, and overall quality of life [18, 19]. On the contrary, poor mental health is strongly tied to poor sleep [15, 16].

During recent years, the level of sleep quality has been globally diminishing in general, and it is a critical determinant of mental health particularly in university students who are susceptible to both mental issues and sleep disturbances [20]. Making a bridge between sleep quality and public health, scholars demonstrated that almost all the aspects of public health such as physical, neurological, and emotional are affected by sleep quality and university students are among the population groups who are at the higher of poor sleep quality [20, 21]. The bidirectionality between sleep disturbances and psychological distress is verified and acknowledged [15, 22], and research traces corroborated the relationship between general mental health and sleep quality in the general population and among university students (particularly medical students) in specific [23–27]. Therefore, the incidence of sleep disturbances and psychological disorders is a predictable outcome, and it would not be out of expectation that significant relationships between sleep quality and depression were conspicuous among medical students [28, 29]. In a nutshell, in order to untie these associations and extend the horizons of implicational understanding, a study interrogating the potential mechanisms in the relationships between sleep quality and mental health is called for [15].

Looking at this picture from another perspective, Almojali et al. demonstrated a significant relationship between stress and poor sleep quality in the medical context, and they considered stress and depression as integral sources in the academic milieu [30]. The issue of sleep quality and psychological factors has been the *focus* of numerous studies; in this regard, previous research indicated that healthcare students are more prone to idiosyncratic stressors, namely, cumbersome courses, lengthy tenure in university, lack of recreation time, and disproportionate self-pressure in their performances in comparison to the counter groups; and consequently, all these factors provide the basis for high levels of depression and poor sleep quality [29, 31].

Despite the affluence of the conducted studies on depression and anxiety among students, paucity of attention has been garnered to freshmen students, and hence, it calls for further and comprehensive investigations. Henceforward, this study centralized its integral aim on investigating the association of depression and anxiety with sleep quality in freshmen students of Neyshabur University of Medical Sciences (NUMS).

## 2. Materials and Methods

In this cross-sectional study, the baseline data of the NUMS student cohort which had its focus on the socioeconomic, physical, and mental properties as well as the educational achievements of the students were used. Participants of this cohort study included all the freshmen students who enrolled in NUMS in 2019 (266 persons) and 2020 (205 persons). The follow-ups started from the beginning of enrollment and would be continued to the ripening time of graduation and following that the baseline data were obtained during September 2019 and 2020. The data collection instruments of the cohort study were checklists and questionnaires designed in an accessible format in which questions about the socioeconomic, demographic, lifestyle, and health characteristics of the students were completed by themselves after obtaining informed consent (including informing the study population about the study's objectives, the procedures, and their rights). The inclusion criteria of the student cohort study consisted of (a) being a newly joined student and (b) consenting to participate in the study.

### 2.1. Dependent Variable

The research population's level of sleep quality was evaluated using the 19-Item Pittsburgh Sleep Quality Index (PSQI), which was validated in the Iranian population by Farrahi Moghaddam et al. [32]. Alimirzaei et al. validated this scale for the Iranian context (students of medical sciences) [33]. The self-rated PSQI questionnaire determines the sleep quality of participants over the past month, and the score of the PSQI ranges from 0 to 21; a lower score shows better sleep quality. A score of more than 5 is considered poor sleep quality. The reported sensitivity and specificity of the PSQI tool were 100% and 93%, respectively.

### 2.2. Independent Variables

The Persian Beck Depression Inventory-II (BDI-II) has been utilized to assess the depression of the study population. Ghassemzadeh et al. validated the Persian version of the scale [34]. It has 21 items, each of which scores from 0 to 3. The total point was calculated by adding up each item's score, ranging from 0 to 63. The total score demonstrated the level of depression, in which the interpretations are as follows: (i) 0-13 represents without depression, (ii) 14-19 represents mild depression, (iii) 20-28 represents moderate depression, and (iv) 29-63 represents severe depression [35]. In this study, three categories of mild, moderate, and severe depression were considered as “yes” and compared with the category of without depression.

Likewise, the Beck Anxiety Inventory (BAI) is used for evaluating anxiety, and it has been validated for the Iranian population by Kaviani and Mousavi [36]. Similar to the depression questionnaire, it has 21 items with scores ranging from 0 to 3 for each item, in which 0 means “not at all,” one means “mildly, but it did not bother me much,” two means “moderately, it was not pleasant at times,” and three means “severely, it bothered me a lot” for all the items. In the same way, the total point varies from 0 to 63, which indicates levels of anxiety. A score from 0 to 7 represents a minimal range (without anxiety). Subsequently, a score of 8 to 15 indicates mild, 16 to 25 represents a moderate range, and further, a score of 26 and above designates severe anxiety. Moreover, compared with the category of without anxiety, the three categories of mild, moderate, and severe anxiety were considered as “yes” [37].

### 2.3. Covariates

In this study, some variables such as sex (female vs. male), age (<20 years old vs. ≥20 years old), marital status (single vs. married), body mass index (BMI) (<25 vs. ≥25), family residency (urban vs. rural), ethnicity (Fars vs. other), family size (<4 vs. ≥4), smoking (no vs. yes), physical activity (low vs. moderate vs. high), and family wealth index (WI) (poorest vs. poor vs. moderate vs. rich vs. richest) were included as covariates.

In this study, the physical activity status of the study population was measured by the International Physical Activity Questionnaire (IPAQ) [38]. According to IPAQ scoring protocol, physical activity was categorized into three categories, including low, moderate, and high. Moreover, the economic status of the student's family was assessed by WI, and it was computed by principal component analysis according to the student's family ownership of some assets.

### 2.4. Statistical Analysis

The variables' frequencies, percentages, means, and standard deviations (SDs) were calculated using descriptive analysis. The association between the study population's sleep quality and their characteristics was evaluated performing the chi-square test. The independent associations of depression and anxiety with sleep quality were assessed using multiple logistic regression model. In order to evaluate the model for significance and model fitness, the Wald and Hosmer-Lemeshow goodness of fit tests were used and the two performed tests passed the associated assumptions as well. All statistical analyses were conducted in STATA^14^, and the significant level was set at *P* < 0.05.

## 3. Results

From the total of 471 studied students, 62.4% were female and the mean age of them was 21.9 ± 6.1. Most of the study population was single (84.7%) and less than a quarter of them had a BMI ≥ 25, and the majority of subjects were nonsmokers (94.7%) as well. Of the study population, 57.4% reported moderate to the richest level of family WI, while the rest of them had poor or poorest level of family WI. Other characteristics of the studied population are presented in Table 1. When it comes to the prevalence of depression and anxiety in the studied students, 78.6% and 68.1% of the students did not show symptoms of depression and anxiety, respectively. However, 21.4% of the students suffered depression, and approximately 32% of the students had anxiety (Table 1).

**Figure 1:**
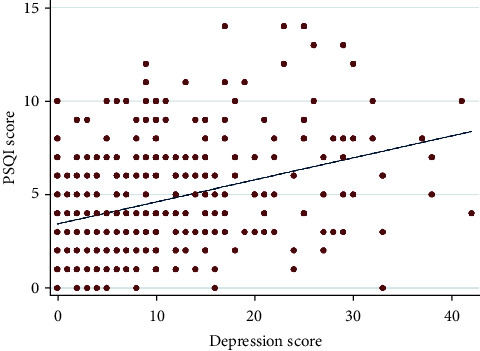
Correlation plot comparing depression scores and sleep quality scores.

**Figure 2:**
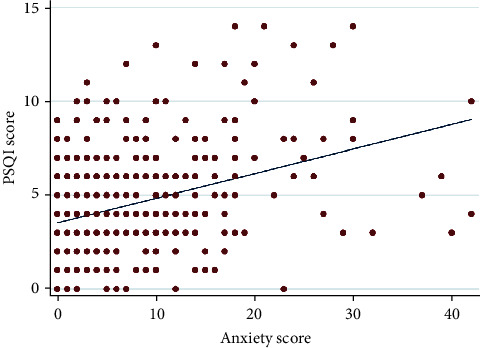
Correlation plot comparing anxiety scores and sleep quality scores.

With respect to poor sleep quality, its prevalence among the study population was 28%.  Table 2 brightly shows the sleep quality categorizations in the study population based on sex, age, marital status, BMI, family residency, family size, smoking, family WI, depression, and anxiety. According to the results of correlation analysis (Pearson's *r*), the correlations between depression and anxiety values with the value of sleep quality were in moderate positive correlations (Figures 1 and 2). Accordingly, Pearson's *r* for depression was 0.38 and Pearson's *r* for anxiety was 0.36.

According to Table 2, there were four variables including sex, smoking, depression, and anxiety (*P* < 0.05) that were in associations with sleep quality (Table 2). At the multiple logistic regression models, the adjusted models for the studied covariates (sex, age, marital status, BMI, family residency, ethnicity, family size, smoking, physical activity, and family WI) showed an independent strong association between depression and sleep quality. Consequently, the results illuminated that the odds of having poor sleep quality in individuals with depression were 3.5 times higher than those without depression (adjusted odds ratio (AOR) = 3.5, 95% confidence interval (CI) = 2.15, 5.70; *P* value < 0.001) (Table 3). Additionally, adjusted analysis has shown that anxiety has an independent association with sleep quality, and hence, the odds of poor sleep quality in the anxious population were 2.1 times higher compared to the nonanxious population (AOR = 2.1, 95% CI = 1.37, 3.37; *P* value = 0.001) (Table 3).

## 4. Discussion

Throughout the society, mental disorders like depression and anxiety are among the most critical crises. Despite great changes in the milieu of medical education, depression remains a critical issue. During the past decade, the trend of mental disorder prevalence has been increasing; in this regard, frequent wars, disasters, and other emergency conditions accompanied by limited psychological services led to worse mental health conditions in low-middle-income countries [39, 40]. In the field of mental health, depression and anxiety have attracted increasing attention particularly in students. From this respect, examining the aspects of students' mental health and its relation with other important health outcomes like sleep quality has become highlighted. Accordingly, the present study has been conducted to interrogate the relationship between depression and anxiety with sleep quality in freshmen students of NUMS.

In the present study, the students reported a higher prevalence of anxiety (32%) compared to that of depression (21%). Similarly, in a study conducted by Ghrouz et al., the prevalence of anxiety in college students was 30% and the prevalence of depression was 18% [40]. Finding more traces, severe depressive symptoms were reported by 15% of Bangladeshi students during the COVID-19 outbreak, while 18% of the students suffered from severe anxiety [41]. Finding a similar recent trace, Carpi and Vestri indicated that on the basis of Hochberg's GT2 post hoc tests, students in the second year had higher levels of anxiety compared to the fifth-year students [28]. One line of justification for our finding can be the fact that transition from high school to university and, meanwhile, age transition as well as living in a new city far from family might increase stress in students. Depression and anxiety can be the consequences of stress, especially in freshmen students [42, 43]. A more vigorous defense might be tied to the fact that universities frequently check the students' mental status on a common consultancy basis [44]. Unlikely, our results held contradictory views with previous research findings in that the Malaysian study reported slightly higher levels of depression (59.2%) than anxiety (55.1%) [45]. Such differences could be due to variations in the use of tools, the structure of the academic university, variant years of data collection, geographical settings and sociocultural contexts, and other variabilities among studies.

More than a quarter of the students reported poor sleep quality in this study. Also, female students, smokers, and those with depression and anxiety were more likely prone to poor sleep quality. In line with our results, Wang et al. revealed that 27.8% of students suffered from poor sleep quality [46]. Likely, poor sleep quality was recorded in 33.7% and 39.5% of Indian and Pakistani students, respectively [47, 48]. Of the limited number of studies of first-year students, in the study of Mamun et al., only 16% of the freshmen students were classified as poor sleepers [49].

Based on multiple logistic regression, it has been identified that the odds of poor sleep quality were higher in the students with depression and anxiety. Similarly, earlier investigations presented the negative effect of depression and anxiety on sleep quality. For example, in a study on Turkish students, the researchers found an association between depression and anxiety with poor sleep quality [50]. A cross-sectional study on students in Nigeria showed that depression and anxiety are strongly associated with poor sleep quality in undergraduate students [51]. Further, a bidirectional association between mental disorders and sleep quality has been found by Alvaro et al. To be more detailed, both depression and anxiety were significant predictors of sleep disturbances and sleep quality and vice versa [15]. According to our results and the above-mentioned studies, it is observed that impaired mental health (such as depression and anxiety) is a substantial predictor of poor sleep quality.

In line with the previous research findings, poor sleep quality is tied to depressive and anxiety symptoms. More importantly, sleep disturbance was found to be a vigorous predictor of subsequent mental disorders and anxiety [52]. Therefore, a thread of evidence now commends that sleep disorders mostly transpire prior to depressive and anxiety symptoms and could be considered a prodromal symptom of future mental disorders. Convenient interventions for sleep disorders may ward off the subsequent consequences of depressive and anxiety symptoms. In harmony with our findings, averting and intervening in sleep disorders might be a golden and cost-effective way to abate the rate of depression and anxiety in medical students [15].

There were some limitations in this study. First, this is a cross-sectional study, and due to its nature, we cannot extract causative findings. Second, the Pittsburgh's instrument subjectively measures sleep quality. Third, recall bias from the study population and use of students from one university rather than several colleges are other limitations. Fourth, with regard to depression and anxiety, the Beck's instruments are only used to screen for symptoms of the mentioned diseases, though the final decision should be left to a medical professional. Fifth, the data of this study might have been affected by other confounding factors; therefore, conducting more studies with more sample size and covariates is recommended. Collapsing three levels of depression and anxiety to “yes” is another limitation of this study.

Under the shade of these limitations, this study enjoys some strengths. In the current study, the effect of various confounding factors had been controlled when we evaluated the association of depression and anxiety with sleep quality with the use of a multiple logistic regression model. Second, we used a representative sample of NUMS students including clinical and nonclinical medical students. In most previous studies, the role of sleep hygiene was assessed as a predictor of mental disorders [53, 54], although the present study is one of the limited studies in which the role of depression and anxiety in relation to sleep quality was examined.

## 5. Conclusion

In conclusion, the results of this study have supported that depression and anxiety are significantly associated with poor sleep quality in freshmen students of NUMs. Perchance, it appears imperative to develop and execute targeted interventional programs aimed at enhancing the mental well-being and sleep quality of medical students. Fulfilling such a significant matter is facilitated through by providing appropriate support and resources to the relevant authorities and stakeholders. Shedding much light, such initiatives could encompass comprehensive strategies for addressing depression and anxiety, as well as promoting better sleep habits and overall wellness within the academic milieu.

## Figures and Tables

**Table 1:** Characteristic of the study population (*n* = 471).

Variables	Number	Percent
Sex		
Female	294	62.4
Male	177	37.6
Age (years)		
<20	276	58.6
≥20	195	41.4
Marital status		
Single	399	84.7
Married	72	15.3
BMI		
<25	361	76.6
≥25	110	23.4
Family residency		
Urban	370	78.6
Rural	101	21.4
Ethnicity		
Fars	404	85.8
Other	67	14.2
Family size		
≤4	186	39.5
>4	285	60.5
Smoking		
No	446	94.7
Yes	25	5.3
Physical activity		
Low	218	46.3
Moderate	135	28.7
High	118	25
Family WI		
Poorest	102	21.7
Poor	98	20.6
Moderate	84	17.8
Rich	93	19.8
Richest	93	19.8
Depression		
No	370	78.6
Yes	101	21.4
Anxiety		
No	321	68.1
Yes	150	31.9

Abbreviations: BMI: body mass index; WI: wealth index.

**Table 2:** Bivariate analyses for the association of studied variables with sleep quality.

	Sleep quality		OR	95% CI	*P* value
	Good 339 (72%)	Poor 132 (28%)			
Sex					
Female	201 (68.4)	93 (31.6)	1	—	—
Male	138 (78.0)	39 (22.0)	0.61	0.40-0.94	0.025
Age					
≤20 years	194 (70.3)	82 (29.7)	1	—	—
>20 years	145 (74.4)	50 (25.6)	0.81	0.54-1.2	0.333
Marital status					
Single	283 (70.9)	116 (29.1)	1	—	—
Married	56 (77.8)	16 (22.2)	0.69	0.38-1.27	0.235
BMI					
<25	259 (71.7)	102 (28.3)	1	—	—
≥25	80 (72.7)	30 (27.3)	0.95	0.59-1.54	0.841
Family residency					
Urban	264 (71.3)	106 (28.7)	1	—	—
Rural	75 (74.3)	26 (25.7)	0.86	0.52-1.42	0.565
Ethnicity					
Fars	288 (71.3)	116 (28.7)	1	—	
Other	51 (76.1)	16 (23.9)	0.78	0.43-1.42	0.416
Family size					
≤4	135 (72.6)	51 (27.4)	1	—	—
>4	204 (71.6)	81 (28.4)	1.05	0.69-1.58	0.813
Smoking					
No	327 (73.3)	119 (26.7)	1	—	—
Yes	12 (48.0)	13 (52.0)	2.97	1.32-6.71	0.008
Physical activity					
Low	154 (70.6)	64 (29.4)	1.72	1.0-2.95	0.05
Moderate	90 (66.7)	45 (33.3)	2.07	1.16-3.69	0.014
High	95(80.5)	23 (19.5)	1	—	—
Family WI					
Poorest	74 (72.5)	28 (27.5)	1	—	—
Poor	67 (68.4)	31 (31.6)	1.2	0.66-2.25	0.517
Moderate	63 (75.0)	21 (25.0)	0.88	0.45-1.70	0.706
Rich	66 (71.0)	27 (29.0)	1.08	0.58-2.02	0.806
Richest	68 (73.1)	25 (26.9)	0.97	0.52-1.83	0.929
Depression					
No	290 (78.4)	80 (21.6)	1	—	—
yes	49 (48.5)	52 (51.5)	3.8	2.42-6.11	<0.001
Anxiety					
No	250 (77.9)	71 (22.1)	1	—	—
yes	89 (59.3)	61 (40.7)	2.4	1.59-3.67	<0.001

Abbreviations: BMI: body mass index; WI: wealth index; OR: odds ratio; CI: confidence interval.

**Table 3:** Multiple logistic regression model for the association of depression and anxiety with sleep quality.

	AOR	95% CI	*P* value
Depression^∗^	3.5	2.15-5.70	<0.001
Anxiety^∗^	2.1	1.37-3.37	0.001

Abbreviations: AOR: adjusted odds ratio; CI: confidence interval. ^∗^Adjusted for sex, age, marital status, BMI, family residency, ethnicity, family size, smoking, physical activity, and family WI.

## Data Availability

The data used to support the findings of this study are available from the corresponding author upon request and after obtaining premission from the institution's ethics committee.
